# Channel Estimation in DCT-Based OFDM

**DOI:** 10.1155/2014/813429

**Published:** 2014-03-13

**Authors:** Yulin Wang, Gengxin Zhang, Zhidong Xie, Jing Hu

**Affiliations:** Institute of Communication Engineering, PLA University of Science and Technology, Yudao Street 14, Nanjing 210007, China

## Abstract

This paper derives the channel estimation of a discrete cosine transform- (DCT-) based orthogonal frequency-division multiplexing (OFDM) system over a frequency-selective multipath fading channel. Channel estimation has been proved to improve system throughput and performance by allowing for coherent demodulation. Pilot-aided methods are traditionally used to learn the channel response. Least square (LS) and mean square error estimators (MMSE) are investigated. We also study a compressed sensing (CS) based channel estimation, which takes the sparse property of wireless channel into account. Simulation results have shown that the CS based channel estimation is expected to have better performance than LS. However MMSE can achieve optimal performance because of prior knowledge of the channel statistic.

## 1. Introduction

MULTICARRIER modulation (MCM) is a promising technique for high data rate transmission. It is used in many digital communications standards, such as local area networks (IEEE 802.11a/g/n), metropolitan area networks (IEEE802.16a), and digital audio and terrestrial video broadcast (DAB/DVB-T) in wireless digital communications systems and such as asymmetric digital subscriber loop (ADSL) in wireline digital communications system. All of these systems belong to the class of discrete Fourier transform- (DFT-) based orthogonal frequency-division multiplexing (OFDM) [1], referred to as DFT-OFDM in this paper.

DFT-OFDM system employs the complex exponential functions set as orthogonal basis. It realizes digital modulations and demodulations by the inverse DFT (IDFT) and DFT, respectively [1]. However, in the literature, [2–5] proposed using a single set of cosinusoidal functions as the orthogonal basis to construct baseband multicarrier signals. This MCM scheme can be synthesized using a discrete cosine transform (DCT). Here, we will denote the scheme as DCT-OFDM.

Reference [3] has shown that DCT-OFDM leads to complete elimination of interblock and intercarrier interference at the same guard sequence overhead, compared with DFT-OFDM. Results in [2] have proved that the bit-error probability (BEP) performance of DCT-OFDM is superior to that of DFT-OFDM in the presence of carrier-frequency offset (CFO), due to the spectral compaction and energy concentration properties of the DCT. Additionally, DCT-OFDM is implemented with a better integrated system design and a reduced overall signal-processing complexity [3].

DCT-OFDM and DFT-OFDM systems are both robust to the multipath induced intersymbol interference (ISI) due to the orthogonal property. However, suffering from the frequency selective fading of the dispersive wireless channel, some subchannels may face deep fading and degrade the overall system performance. On the other hand, multipath can also bring multiplexing/diversity gains which improve the rate/reliability of communication system. As a result, one has to perform accurate channel estimation to obtain channel state information (CSI) before coherent demodulation.

The channel estimation for DFT-OFDM systems has been studied by many researchers [6–9]. However, there is still no investigation on channel estimation for DCT-OFDM system. On the other hand, the radio channel in a wireless communication system is often characterized by multipath propagation, typically with a few distinct paths, resulting in a sparse multipath channel model [10]. A recent advance in compressed sensing (CS) [11] provides a potential solution to reducing the required number of pilot symbols. Compressed channel sensing (CCS) was proposed in [12] for a frequency selective channel. Reference [13] applied distributed compressive sensing (DCS) theory to a channel estimation application. An efficient pilot design scheme was studied in [14] for seeking optimal pilot placement for sparse channel estimation. In [15], authors designed low-complexity sparse channel estimation and tracking method for time-varying DFT-OFDM channels.

In this paper, we design channel estimation methods for DCT-OFDM system. They are, respectively, least square (LS) estimator, mean square error estimator (MMSE), and compressed sensing estimator (CSE). The CSE directly uses DCT matrix as the orthogonal sparse dictionary. Compared with the two conventional estimators, CSE exploits the inherent sparse property of channels, saving pilots symbols for the same estimation performance. Thereby, CSE leads to economize the key resources, such as energy, latency, and bandwidth in DCT-OFDM system.

The remainder of this paper is organized as follows. In Section 2, we construct the DCT-OFDM system and give the system model. Then we introduce the LS and MMSE estimator for DCT-OFDM system in Section 3.  Section 4 gives a brief review of CS theory and its application to sparse channel estimation in DCT-OFDM. In Section 5, we succinctly summarize the performance of the three estimators. We devote the conclusions in Section 6.

## 2. Signal Model

**Figure 1:**
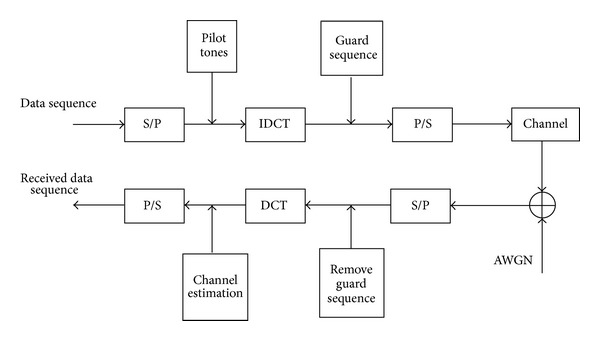
DCT-based OFDM systems.

Figure 1 has shown a DCT-OFDM system, in which modulation/demodulation is done by IDCT/DCT. Unlike conventional DFT-OFDM, a single cosinusoidal functions set cos⁡(2*πnF*_Δ_ *t*), *n* = 0,1,…, *N* − 1, will be used as the orthogonal basis to implement MCM in DCT-OFDM system. The minimum *F*_Δ_ required to satisfy

(1) $$\begin{array}{c} \overset{T}{\underset{0}{\int }}\sqrt{\frac{2}{T}}\cos\left(2\pi kF_{\Delta }t\right)\sqrt{\frac{2}{T}}\cos\left(2\pi mF_{\Delta }t\right)dt=\{\begin{array}{cc} 1, & k=m\\ 0, & k\neq m \end{array} \end{array}$$

is 1/2*T* Hz. The continuous-time representation of a baseband DCT-OFDM block *x*(*t*) is

(2) $$\begin{array}{c} x\left(t\right)=\sqrt{\frac{2}{N}}\overset{N-1}{\underset{n=0}{\sum }}d_{n}\beta_{n}\cos\left(\frac{n\pi t}{T}\right), \end{array}$$

where *d*_0_, *d*_1_,…, *d*_*N*−1_ are *N* independent data symbols obtained from a modulation constellation, and

(3) $$\begin{array}{c} \beta_{n}=\{\begin{array}{cc} \frac{1}{\sqrt{2}}, & n=0\\ 1, & n=1,2,\ldots ,N-1. \end{array} \end{array}$$

Sampling the continuous-time signal *x*(*t*) at time instants *t*_*m*_ = *T*(2*m* + 1)/2*N* gives a discrete time sequence [16]:

(4) $$\begin{array}{c} x_{m}=\sqrt{\frac{2}{N}}\overset{N-1}{\underset{n=0}{\sum }}d_{n}\beta_{n}\cos\left(\frac{\pi n\left(2m+1\right)}{2N}\right),\;m=0,1,\ldots ,N-1 \end{array}$$

which is the inverse DCT (IDCT). Thus, the continuous-time signal *x*(*t*) can be obtained by first performing an IDCT operation on data sequence:

(5) $$\begin{array}{c} d={\left[d_{0},d_{1},\ldots ,d_{N-1}\right]}^{T}, \end{array}$$

where []^*T*^ represents the transpose operation and then feeds serially the resulting samples **x** = [*x*_0_,*x*_1_,…,*x*_*N*−1_]^*T*^ through a digital-to-analog (D/A) converter.

Signal is transmitted through a frequency-selective multipath fading channel. We assume the channel impulse response (CIR) is constant during one DCT-OFDM symbol. At the receiver sketched in Figure 1, after matched filtering, the signal is sampled at rate 1/*T*_*s*_ and serial to parallel converted. We indicate with **h** = [*h*_0_,*h*_1_,…,*h*_*L*−1_]^*T*^ the *T*_*s*_-spaced samples of the overall CIR:

(6) $$\begin{array}{c} y=x⊗h+w, \end{array}$$

where ⊗ denotes cyclic convolution and **w** is additive white Gaussian noise (AWGN) with zero mean and variance *σ*_*w*_^2^.

Denoting

(7) $$\begin{array}{c} H_{n}=\sqrt{\frac{2}{N}}\overset{N-1}{\underset{k=0}{\sum }}h_{k}\cos\left(\frac{\pi k\left(2n+1\right)}{2N}\right),\;n=0,1,\ldots ,L-1 \end{array}$$

the DCT of **h**, and **H** = [*H*_1_, *H*_2_,…, *H*_*L*−1_] is the channel response in DCT domain also called the channel frequency response in this paper. After removing the guard sequence, the received samples are passed to an *N*-point DCT unit. The output of the DCT unit is found to be

(8) $$\begin{array}{c} r_{n}=d_{n}H_{n}+{\tilde{w}}_{n}, \end{array}$$

where *r*_*n*_ is the received signal and ${\tilde{w}}_{n}$ is noise in DCT domain.

In this study, we assume that some known symbols (pilots) are multiplexed into the data stream, and channel estimation is performed at pilots locations. A total of *N*_*p*_ pilots {*c*_*n*_; 0 ≤ *n* ≤ *N*_*p*_ − 1} are inserted in one DCT-OFDM block at known locations {*i*_*n*_; 0 ≤ *n* ≤ *N*_*p*_ − 1}. The *N*_*p*_-dimensional vector containing the DCT output at the pilot locations is denoted by **r** = [*r*_0_,*r*_1_,…,*r*_*N*_*p*_−1_]^*T*^; from (7) and (8), we have

(9) $$\begin{array}{c} r=DFh+\tilde{w}=DH+\tilde{w}, \end{array}$$

where $\tilde{w}=\left[{\tilde{w}}_{1},{\tilde{w}}_{2},\ldots ,{\tilde{w}}_{L-1}\right]$ is the AWGN noise vector in DCT domain and **D** is a diagonal matrix containing pilot symbols

(10) $$\begin{array}{c} D=\mathrm{diag}\left\{c_{0},c_{1},\ldots ,c_{N_{p}-1}\right\} \end{array}$$

and **F** is an *N*_*p*_ × L DCT matrix with entries:

(11) $$\begin{array}{c} F\left(k,n\right)=\sqrt{\frac{2}{N}}\beta_{k}\cos\left(\frac{\pi k\left(2n+1\right)}{2N}\right). \end{array}$$

## 3. LS and MMSE Channel Estimation

A conventional approach to estimate the channel is the least square (LS) estimation of channel frequency response on the pilot subcarriers. Then, the frequency domain LS estimation of CIR can be denoted as

(12) $$\begin{array}{c} {\hat{H}}_{\text{LS}}=D^{†}r=H+D^{†}w, \end{array}$$

where † denotes the pseudoinverse of a matrix and the noise power of LS estimation is calculated as tr⁡{*σ*_*w*_^2^(**D** ^†^)^*H*^ **D** ^†^}, and tr⁡{·} means the trace of a matrix.

Corresponding to [6], if the channel vector **h** is Gaussian and uncorrelated with the channel noise **w**, the MMSE estimate of ${\hat{h}}_{\text{MMSE}}$ could be obtained by

(13) $$\begin{array}{c} {\hat{h}}_{\text{MMSE}}=R_{hr}R_{rr}^{-1}r, \end{array}$$

where

(14) $$\begin{array}{c} R_{hr}=E\left\{hr^{H}\right\}=R_{hh}F^{H}D^{H},\\ R_{rr}=E\left\{rr^{H}\right\}=DFR_{hh}F^{H}D^{H}+\sigma_{w}^{2}I_{N}, \end{array}$$

where **R**_**hr**_, **R**_**rr**_, and **R**_**hh**_ are, respectively, the cross covariance matrix between **h** and **r**, the autocovariance matrix of **r**, and the autocovariance matrix of **h**. Noise variance *σ*_*w*_^2^ and **R**_**hh**_ are assumed to be known. The frequency domain estimate vector ${\hat{H}}_{\text{MMSE}}$ is given by

(15) $$\begin{array}{c} {\hat{H}}_{\text{MMSE}}=F{\hat{h}}_{\text{MMSE}}. \end{array}$$

Both estimators (12) and (15) have their drawbacks. The LS estimator has low complexity, but it performs a high mean square error. The MMSE estimator has its perfect performance. However, it suffers from a high complexity and needs to know the statistics of the channel (*σ*_*w*_^2^ and **R**_**hh**_) as a prior information. This condition is usually impossible in practical engineering.

## 4. CS Based Channel Estimation

### 4.1. Compressed Sensing Overview

The CS principles solve the problem of exactly reconstructing an *N* × 1 signal from with a small portion of linear measurements. Consider an *N* × 1 vector **x**, which can be represented as ***θ*** in some orthogonal basis *ψ* = [*ψ*_1_, *ψ*_2_,…, *ψ*_*N*_]:

(16) $$\begin{array}{c} x=𝚿\theta . \end{array}$$

***θ*** is a *K*-sparse representation of **x**, where *K* ≪ *N*, because only *K* coefficients of ***θ*** are not zero. From CS theory, **x** can be recovery from a linear measurement vector:

(17) $$\begin{array}{c} y=𝚽x=𝚽𝚿\theta , \end{array}$$

where *𝚽* is an *M* × *N* (*M* < *N*) observation matrix satisfying Restricted Isometry Property (RIP).

*Restricted Isometry Property* [17]: the observation matrix *𝚽* is said to satisfy the restricted isometry property of order *S* with parameter *δ*_*S*_ ∈ (0,1), if

(18) $$\begin{array}{c} \left(1-\delta_{S}\right){||z||}_{l_{2}}^{2}\leq {||𝚽z||}_{l_{2}}^{2}\leq \left(1+\delta_{S}\right){||z||}_{l_{2}}^{2} \end{array}$$

holds for all *S*-sparse vectors **z** ∈ **R** ^*n*^.

If there is no noise in observation **y**, the reconstruction problem can be solved by an *l*_1_-norm optimization:

(19) $$\begin{array}{c} \hat{\theta }=\arg\underset{\theta }{\min}{||\theta ||}_{1}\;\;\text{s}.\text{t}.\;\;\;y=𝚽𝚿\theta . \end{array}$$

Basis pursuit (BP) is appropriate to solve the basis pursuit problem. If the observation **y** is contaminated with noise, then an additional norm of the residual *𝚽𝚿* ***θ*** − **y** should be minimized as

(20) $$\begin{array}{c} \hat{\theta }=\arg\underset{\theta }{\min}{||\theta ||}_{1}+\frac{\mu }{2}{||𝚽𝚿\theta -y||}_{2}^{2} \end{array}$$

or constrained

(21) $$\begin{array}{c} \hat{\theta }=\arg\underset{\theta }{\min}{||𝚽𝚿\theta -y||}_{2}^{2}\;\;\text{s}.\text{t}.\;\;\;{||\theta ||}_{1}\leq \eta , \end{array}$$

where *μ* and *η* are constants. Formulation (20) is the *l*_1_-regularized least squares problem and (21) is the least absolute shrinkage and selection operator (Lasso) problem [18]. On the other hand, there is also a greedy algorithm called Orthogonal Matching Pursuit (OMP) [19] to handle the vector recovery problem. Such method iteratively selects the local optimal solution step by step. The major advantages of OMP algorithm are its ease of implementation and its speed.

### 4.2. CS Based Channel Estimation in DCT-OFDM

Compare (9) with (17), we can find that channel estimation for DCT-OFDM system is able to be settled by CS theory. However, different from CSE in the existed literature, the orthogonal basis in our system model is DCT matrix other than DFT matrix:

(22) $$\begin{array}{c} r=DFh+w=DH+w, \end{array}$$

where pilot data matrix **D** represents as the measure matrix, DCT matrix **F** acts as the sparse dictionary, and **r** is the measure vector. Thus, pilot-aided channel estimation has been formulated as a sparse reconstruction problem discussed above. The computation feasible takes advantage of available fast transform algorithms for DCT [20].

Similar to (21), CIR could be obtained by solving

(23) $$\begin{array}{c} {\hat{h}}_{\text{CSE}}=\arg\underset{h}{\min}{||DFh-r||}_{2}^{2}\;\text{s}.\text{t}.\;{||h||}_{1}\leq \eta . \end{array}$$

The frequency domain estimate vector ${\hat{H}}_{\text{CSE}}$ is given by

(24) $$\begin{array}{c} {\hat{H}}_{\text{CSE}}=F{\hat{h}}_{\text{CSE}}. \end{array}$$

Comparing to the LS and MMSE channel estimation, CSE exploits the inherent sparse property of multipath channels. Thereby, CSE leads to economizing the key communication resources of DCT-OFDM system, such as energy, latency, and bandwidth [10].

## 5. Simulation Results

In this section, we present the simulation results to compare the performance of the proposed LS, MMSE, and CS-based channel estimation methods for DCT-OFDM system. The channel **h** is assumed to have *L* = 42 taps. However, only *K* = 10 taps have nonzero values and their positions are randomly distributed. The number of DCT-OFDM subcarriers is 256. Date sequences are modulated by binary phase shift keying (BPSK).

**Figure 2:**
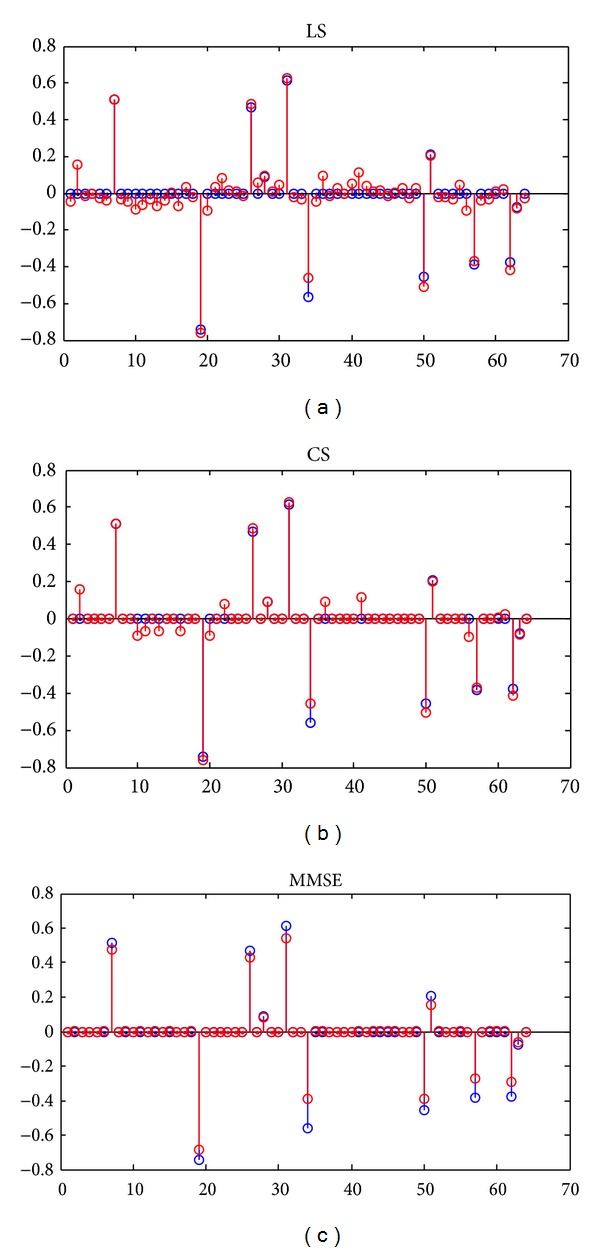
Channel estimated by LS, CS, and MMSE estimation.

Figure 2 shows channel taps estimated by LS estimation, CS based estimation, and MMSE estimation, respectively. Blue taps are original channel taps. The estimation parameters are SNR = 10 dB, pilot symbols are modulated by BPSK, and pilots number *N*_*p*_ = 30. Channel estimated by CS based estimation is much clearer and less noise contaminated than LS estimation. Channel estimated by MMSE is the clearest and almost no noise contaminated.

**Figure 3:**
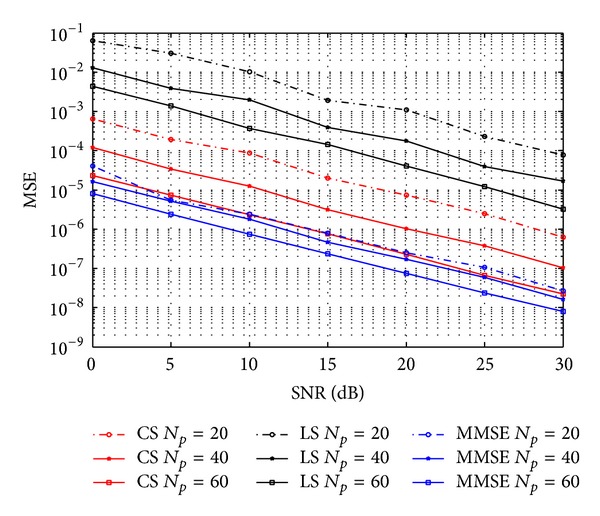
MSE performance of LS, CS, and MMSE estimation.

Figure 3 presents the mean square error (MSE) versus the signal-to-noise ratio (SNR). We select the number of pilots to be *N*_*p*_ = 14,28, and 42. For LS estimation, when number of pilot symbols is less than channel length *L*, performance of estimation is terrible. However, it is seen that the CS estimation significantly outperforms the LS estimation. Channel estimated by CS with pilots much less than LS is more accurate than LS. This phenomenon means that CS can save bandwidth and energy compared with LS. On the other hand, MMSE performs best due to its prior knowledge of channel statistic mentioned in Section 3. This is hardly possible in practical application.

**Figure 4:**
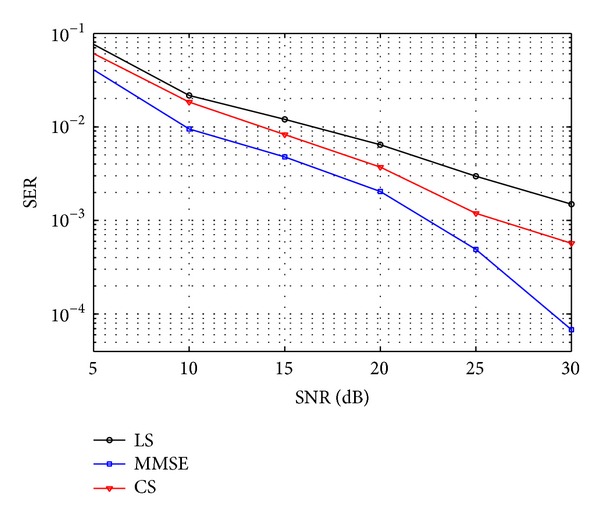
SER performance of LS, CS, and MMSE estimation.

Figure 4 plots the symbol error rate (SER) curves versus SNR. Channel estimations are done under the condition that pilot symbols are modulated by BPSK and pilots number *N*_*p*_ = 30. We can find that CS channel estimation performs much better than LS. MMSE is always the best among the three methods.

**Figure 5:**
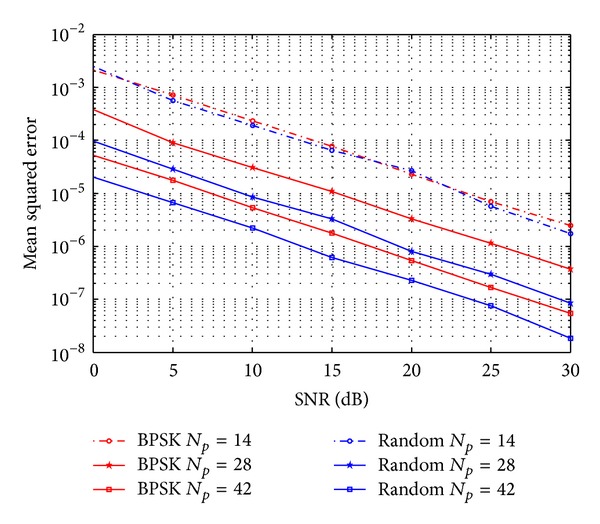
MER performance of CS channel estimation for different kind of training sequence.

Figure 5 presents the MER curves that compare channel estimation performance for CSE using different classes of pilot symbols. Red curves represent random generated symbols, and blue curves represent BPSK modulated pilots. In the case of *N*_*p*_ = 14, performances of the two kinds of pilots seem pretty much the same thing. But performance of random modulated pilots is much better in the other two cases.

## 6. Conclusions

In this paper, we have investigated cannel estimation in DCT-OFDM system. We have compared performance of LS, MMSE, and CS based estimation. CS has exploited the sparse property of multipath channel. Compared with LS estimation, CS estimation enables accurate channel estimation with less pilots. This means that CS estimation economizes the key communication resources of DCT-OFDM system, such as bandwidth and energy. Hence, CS channel estimation leads to high throughput and data rate. On the other hand, MMSE can give the optimal channel estimation on the condition of knowing channel statistic as prior information. This condition is unachievable in practical engineering. In our future work, we will focus on CCS scheme in other communication systems.
